# ICTV Virus Taxonomy Profile: Bromoviridae

**DOI:** 10.1099/jgv.0.001282

**Published:** 2019-06-13

**Authors:** Joseph Bujarski, Donato Gallitelli, Fernando García-Arenal, Vicente Pallás, Peter Palukaitis, M. Krishna Reddy, Aiming Wang, ICTV Report Consortium

**Affiliations:** 1 Department of Biological Sciences, Northern Illinois University, IL 60115, DeKalb, USA; 2 Dipartimento di Scienze del Suolo della Pianta e degli Alimenti, Università degli studi di Bari Aldo Moro, 70126, Bari, Italy; 3 Centro de Biotecnología y Genómica de Plantas UPM-INIA, Universidad Politécnica de Madrid, 28223 Pozuelo de Alarcón, Madrid, Spain; 4 Institute for Plant Molecular and Cell Biology, Polytechnic University of València-CSIC, 46011 València, Spain; 5 Department of Horticultural Sciences, Seoul Women’s University, Seoul 01797, South Korea; 6 Division Plant Pathology, Indian Institute of Horticultural Research, Bengaluru, Karnataka, India; 7 London Research and Development Centre, Agriculture and Agri-Food Canada, London, Ontario N5V 4T3, Canada

**Keywords:** ICTV Report, Taxonomy, Bromoviridae

## Abstract

*Bromoviridae* is a family of plant viruses with tri-segmented, positive-sense, single-stranded RNA genomes of about 8 kb in total. Genomic RNAs are packaged in separate virions that may also contain subgenomic, defective or satellite RNAs. Virions are variable in morphology (spherical or bacilliform) and are transmitted between hosts mechanically, in/on the pollen and non-persistently by insect vectors. Members of the family are responsible for major disease epidemics in fruit, vegetable and fodder crops such as tomato, cucurbits, bananas, fruit trees and alfalfa. This is a summary of the International Committee on Taxonomy of Viruses (ICTV) Report on the family *Bromoviridae,* which is available at www.ictv.global/report/bromoviridae.

## Virion

**Fig. 1.**
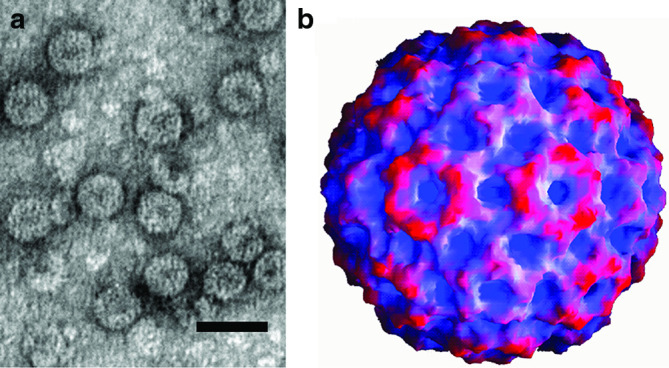
Cucumber mosaic virus particles. (**a**) Negative-contrast electron micrograph (courtesy of A. De Stradis, IPSP-CNR, Bari, Italy) and (**b**) reconstruction (courtesy of Dr K.L. Perry, Cornell University, Ithaca, NY, USA, Dr T. Smith, University of Texas, Galveston, Texas, USA and A. Paredes, NCTR/ORA, Arkansas USA). Bar, 50 nm.

Virions are either spherical or quasi-spherical (Table 1, Fig. 1), having *T*=3 icosahedral symmetry and a diameter of 26–35 nm (genera *Anulavirus*, *Bromovirus*, *Cucumovirus* and *Ilarvirus*), or bacilliform (genera *Alfamovirus*, *Ilarvirus* and *Oleavirus*) with dimensions of 18–26 nm by 30–85 nm. Genomic RNAs are packaged in separate virions that may also contain subgenomic, defective or satellite RNAs [1].

**Table 1.** Characteristics of members of the family *Bromoviridae*

Typical member:	brome mosaic virus, Russian wheat (RNA1: X02380; RNA2: X01678; RNA3: J02042), species *Brome mosaic virus*, genus *Bromovirus*
Virion	Spherical or quasi-spherical (26–35 nm diameter) or bacilliform (18–26 nm by 30–85 nm)
Genome	Three segments of linear positive-sense, single-stranded RNA, comprising about 8 kb in total
Replication	On cytoplasmic membranes with genomic RNAs acting as mRNAs. Coat protein may be required for genome activation
Translation	Directly from genomic or subgenomic RNA
Host range	From narrow to broad range of plants
Taxonomy	Realm *Riboviria*, six genera, including >30 species

## Genome

**Fig. 2.**
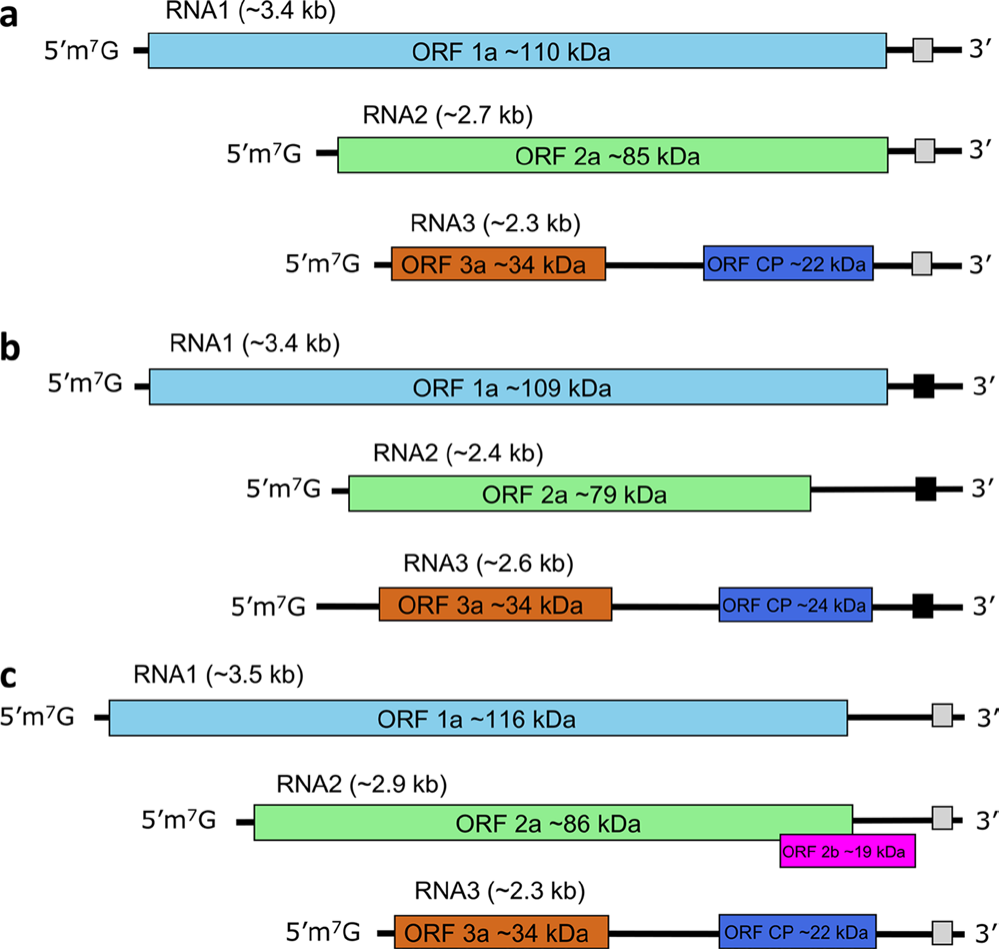
Schematic genome organization for members of the family *Bromoviridae*: (**a**) genera *Alfamovirus, Bromovirus, Ilavirus* subgroups 3 and 4 and *Oleavirus*. (**b**) genus *Anulavirus*. (**c**) genera *Cucumovirus* and *Ilarvirus* subgroups 1 and 2. The 3′-termini form either tRNA-like (**b**) or complex structures (**a, c**) shown as black or grey square boxes, respectively.

The genome of approximately 8 kb is split among three linear, positive-sense ssRNAs with 5′-terminal cap structures. The 3′-termini form tRNA-like or other structures that can be aminoacylated (genera *Bromovirus* and *Cucumovirus*) or not (genera *Alfamovirus*, *Anulavirus*, *Ilarvirus* and *Oleavirus*) (Fig. 2).

## Replication

Replication of genomic and subgenomic RNAs occurs on cytoplasmic membranes via full length negative-sense strand synthesis and subsequent positive-sense strand synthesis. Coat protein may be required for activation of replication (*Alfamovirus* and *Ilarvirus*) whereas a cytoplasmic/nuclear balance of coat protein accumulation modulates viral gene expression (*Alfamovirus*) [1].

## Pathogenicity

Alfalfa mosaic virus (genus *Alfamovirus*) infects many herbaceous and some woody hosts inducing systemic mottling and ‘calico’ mosaic.

Pelargonium zonate spot virus (genus *Anulavirus*) infects tomato plants, that display stunting, concentric chlorotic or necrotic rings and line patterns on leaves, stems and fruit [2].

Members of the genus *Bromovirus* infect some Poaceae or Fabaceae inducing mosaic, brown streaks and reduced seed yield.

Cucumber mosaic virus (genus *Cucumovirus*) exists as many strains, some supporting a 330–390 nt satellite RNA that may induce necrosis in tomato, chlorosis in tomato, tobacco and pepper or attenuate disease symptoms. Hosts include fruit crops, vegetables, ornamentals and weeds [3].

Members of the genus *Ilarvirus* infect fruit trees and some herbaceous crops. Prunus necrotic ringspot virus and prune dwarf virus cause stunting and necrotic lesions on the leaves of sweet cherry, sour cherry, plum and peach trees [4].

Olive latent virus 2 (genus *Oleavirus*) has been recorded in olive and in castor bean. Infections are asymptomatic in olive but produce a yellowish vein netting and mottling of the leaves of castor bean plants [5].

## Taxonomy

The six genera are based on virus host range, genome content and vector. Members of the genera *Alfamovirus* and *Cucumovirus* are transmitted by aphids, those of *Anulavirus* and *Ilarvirus* by thrips and/or pollen, members of *Bromovirus* by beetles, while the transmission route for members of the genus *Oleavirus* is unknown.

### Resources

Full ICTV Report on the family *Bromoviridae*: www.ictv.global/report/bromoviridae.
